# Skip Re-Entry Trajectory Detection and Guidance for Maneuvering Vehicles

**DOI:** 10.3390/s20102976

**Published:** 2020-05-24

**Authors:** Hongqiang Sun, Shuguang Zhang

**Affiliations:** School of Transportation Science and Engineering, Beihang University, Beijing 100191, China; hqsun@buaa.edu.cn

**Keywords:** skip re-entry detection, sensor system enhancement, abnormal re-entry, trajectory control

## Abstract

The re-entry trajectory of maneuvering vehicles with medium to high hypersonic lift-to-drag ratios is generally planned using quasi-equilibrium flight conditions known from Space Shuttles. They may exhibit an oscillation re-entry phenomenon termed skip re-entry when related components or sensors fail. However, conventional re-entry guidance only considers quasi-equilibrium flights and ignores the possibility of the occurrence of an unexpected skip trajectory; this may lead to the failure of the re-entry mission due to a lack of a corresponding guidance strategy. However, the detection of a skip trajectory is the necessary reference for the decision-making of calling a related guidance algorithm that helps improve the safety of vehicle re-entry. Herein, a skip re-entry detection and trajectory control solution is proposed to play an emergency role in the cases of skip re-entry. Firstly, the oscillation frequency characteristics of the linearized re-entry motion equation of a vehicle are analyzed, and an approximate analytical relationship is constructed for skip altitude estimation. Then, the residual deviation between the altitude feedback data and the estimated skip altitude is calculated and compared with the threshold to determine the occurrence of skip re-entry. In addition, a method for controlling the skip re-entry trajectory with the range extension is developed by controlling the bank angle with a fixed angle of attack profile, satisfying the path constraint requirements. The results indicate that the method effectively performs skip re-entry detection and that it can help extend the range of the vehicles in abnormal re-entry scenarios, keeping the flight within the path constraints and guiding it to the expected location.

## 1. Introduction

Experiences of the Space Shuttle provide a baseline for planning and optimizing the re-entry trajectory for vehicles with similar symmetric configurations and medium or high lift-to-drag ratios [1,2]. From low Earth orbit (LEO) back into the atmosphere, the drag-acceleration profile planning algorithm [3] is explicit and practical. Several research studies [4,5,6,7,8,9] are also based on the planning of drag-acceleration profiles, for which the acceleration sensor plays an indispensable role. However, electromagnetic radiation in the near-earth space environment is very complex [10] and may cause sensor performance degradation or failure [11]. However, sensor performance degradation is not easy to detect directly and will lead to a deviation of the result of the guidance algorithm calculation, which may lead to an abnormal re-entry trajectory.

For the improved safety and probability of success, different deviations and abnormal scenarios need to be taken into consideration during vehicle development. For commercial space launch and re-entry activities, the Federal Aviation Administration requires that the trajectory analysis for both normal and malfunction flights must characterize variability and include deviation in paragraphs 450.117 and 119 of 14 CFR (Code of Federal Regulations) Part 450. From LEO re-entry, the trajectory of maneuvering vehicles with medium to high hypersonic lift-to-drag ratios is generally planned by employing quasi-equilibrium flight conditions known from the Space Shuttle [3]. In addition, there are different planning techniques to adapt to various scenarios. Shen et al. provided a rapid generation method of three degrees of freedom (3-DOF) re-entry [12]. Subsequently, Lu provided a unified re-entry guidance method, independent of the hypersonic lift-to-drag ratio (L/D) of the vehicle, which can range from 0.28 for a capsule crew exploration vehicle to 3.5 for a common aero vehicle [13], basically all flying in quasi-equilibrium glide mode. Another possibility, however, is to fly along non-equilibrium trajectories, such as skips that may occur under abnormal conditions.

The skip re-entry trajectory planning was first introduced for the Apollo lunar mission to achieve a long downrange from the orbits; this was done to provide diversion capability to avoid bad and disruptive weather conditions [14]. Orion is also designed with the capability of performing a skip entry for extending the down range [15]. For the skip entry guidance theory, Loh [16,17] proposed a first and second order method to provide an analytical approximate solution, and then Vinh et al. [18] refined and further developed the second order solution for lifting skip trajectories.

For re-entry vehicles, other skipping strategies, such as reference-following controllers [19,20] and numeric predictor–corrector skip re-entry algorithms have been proposed to provide onboard real-time trajectory generation and guidance [21,22]. Furthermore, Brunner and Lu [23,24] integrated trajectory prediction with closed-loop correcting guidance using the bank angle determined based on the downrange requirement. Subsequently, Luo et al. [25,26] presented a skip guidance algorithm that used a numerical predictor–corrector and a patched corridor for low-lifting capsules returning from the Moon, where the distribution of the bank angle was piecewise and linear with respect to the normalized energy. Cheng et al. [27] proposed a numerical multi-constrained predictor–corrector guidance algorithm, which designed a bank corridor to help convert the trajectory planning into a root-finding problem and developed a constraint management module to improve the satisfaction of path and terminal constraints. Liu et al. [28] represented an attempt to apply a second order cone programming, a branch of convex optimization, to a class of highly nonlinear trajectory optimization problem in entry flight. In addition, Wang et al. [29] developed the convex optimization methods to solve hypersonic trajectory optimization problems, which generated a reference trajectory by solving a second order cone programming problem and designed an optimal feedback guidance law using a constrained quadratic programming method to track the trajectory. Moreover, skipping guidance is also closely related to aerocapture, such as the analytic predictor–corrector guidance, which is being discussed for the Mars Sample Return Orbiter vehicle [30], the numerical optimal predictor–corrector guidance algorithms that are being developed to guide the spacecraft through aerocapture into a target orbit [31,32].

For re-entry of a maneuvering vehicle, if only conventional re-entry trajectory guidance is considered and the possibility of skip trajectory is ignored, there may be a loss of control during re-entry due to a lack of any corresponding guidance strategy. During the process of LEO re-entry, sensor data plays a key role in the guidance algorithm, and its failure or performance degradation may lead to a skip trajectory. On one hand, during the process of initial re-entry, the sensor data provides a judgment basis for using different guidance algorithms [13,14,23,24]. When a failure or the performance degradation of any sensor leads to a system misjudgment, the guidance instructions will follow the initial descent instructions. Then, the vehicle goes to a skip mode due to its natural phugoid oscillatory tendency in a dense atmosphere [14] and experiences serious deviation from the original trajectory planning. On the other hand, a quasi-equilibrium glide trajectory planning strategy is widely used for re-entry [12,13]. In this method, the bank angle is normally controlled to suppress the occurrence of a skip; however, any sensor performance degradation may lead to a guidance output which does not restrain the skip, and this may lead to a failure of the guidance algorithm in the skip flight.

It can be seen that, when there is a skip in a routine re-entry process, calling on the corresponding skip trajectory guidance strategy will help with improving the safety of the re-entry vehicle, and the detection of a skip trajectory will be a necessary reference for decision-making when calling the guidance algorithm.

However, conventional sensors used in skip re-entry detection provide only basic data and fail to further determine whether the vehicle will undergo skip re-entry. In addition, for the above skip guidance algorithms, which are mainly employed for expected skip guidance scenarios, such as aerocapture [30,31,32], and extended range entry [15], there is no skip trajectory detection and transition logic, and they are mainly focused on vehicles with L/D ratios below 0.5 at re-entry and velocities close to the escape velocity of the Earth, that is, 11.2 km/s [19,20,21,22,23,24,25,26,30]. However, for the LEO re-entry, a conventional and practical approach for re-entry is to glide in a quasi-equilibrium condition. On this basis, this paper considers an unexpected skip occurrence and expects to monitor or assist decision-making through skip trajectory detection and calls on the corresponding skip guidance algorithm to improve the safety of LEO re-entry.

Therefore, this article proposes a skip re-entry detection and trajectory control solution to deal with the skip problem in trajectory control during the normal re-entry of hypersonic maneuvering winged vehicles with a medium or high L/D.

First, the oscillation frequency characteristics of the linearized equation of motion of the vehicle re-entry are analyzed, and an approximate relationship between the spatial frequency and altitude is obtained. Second, the residual deviation between the altitude feedback data and the estimated skip altitude is calculated and compared with a threshold value to determine whether the vehicle will undergo skip re-entry. In addition, a skip re-entry trajectory control method with the range extension, which is realized by controlling the bank angle of a profile with a fixed angle of attack, and a numerical search algorithm are discussed. Finally, the performance is assessed by skip re-entry detection rate tests, false alarm rate tests and Monte Carlo dispersion simulations.

## 2. Equations and Constraints of Re-Entry Flight

For re-entry, the 3-DOF equations, including the Earth’s curvature and rotation [33], are widely accepted, as follows:

(1) $$\left\{ \begin{array}{l} \dot{r}=V\sin\gamma \\ \dot{\theta }=\frac{V\cos\gamma \sin\psi }{r\cos\phi }\\ \dot{\phi }=\frac{V\cos\gamma \cos\psi }{r}\\ \dot{V}=-\frac{D}{m}-g\sin\gamma +\Omega_{e}^{2}r\cos\phi (\sin\gamma \cos\phi -\cos\gamma \sin\phi \cos\psi )\\ \dot{\gamma }=\frac{1}{V}[\frac{L\cos\sigma }{m}-g\cos\gamma +\frac{V^{2}\cos\gamma }{r}+2\Omega_{e}V\cos\phi \sin\psi +\Omega_{e}^{2}r\cos\phi (\cos\gamma \cos\phi +\sin\gamma \cos\psi \sin\phi )]\\ \dot{\psi }=\frac{1}{V}[\frac{L\sin\sigma }{m\cos\gamma }+\frac{V^{2}}{r}\cos\gamma \sin\psi \tan\phi -2\Omega_{e}V(\tan\gamma \cos\psi \cos\phi -\sin\phi )+\frac{\Omega_{e}^{2}r}{\cos\gamma }\sin\psi \sin\phi \cos\phi ] \end{array}\right.$$

where r is the radial distance from the center of Earth to the vehicle, normalized by the radius of the Earth $r_{e}=6378\;\mathrm{km}$, θ and ϕ are the longitude and latitude, respectively, γ is the flight path angle relative to the surface of the Earth, ψ is the course angle describing the relative velocity vector measured clockwise from the north, σ is the bank angle measured positive to the right from the view inside the vehicle, V is the velocity relative to Earth, m is the mass of the vehicle, g is the gravitational acceleration, $\Omega_{e}$ is the rotation rate of Earth, L and D are aerodynamic lift and drag, respectively, as expressed below:

(2) $$L=\frac{1}{2}C_{L}\rho V^{2}S_{ref},D=\frac{1}{2}C_{D}\rho V^{2}S_{ref}$$

where ρ is the local atmospheric density used in this study, which is in accordance with the 1976 U.S. standard atmosphere model [34], $C_{L}$ and $C_{D}$ are lift and drag coefficients, respectively, $S_{ref}$ is the reference area of the vehicle.

In re-entry, the path constraints often include limits of heat rate, normal load factor, dynamic pressure, typically described as [13]

(3) $$\dot{Q}=C_{Q}\sqrt{\rho }V^{3.15}\leq {\dot{Q}}_{\max}$$

(4) $${\bar{N}}_{Z}=\left|\frac{L}{mg}\cos\alpha +\frac{D}{mg}\sin\alpha \right|\leq n_{\max}$$

(5) $$\bar{q}=\frac{1}{2}\rho V^{2}\leq {\bar{q}}_{\max}$$

where $\dot{Q}$ is the heat rate at a stagnation point on the surface of the vehicle, ${\dot{Q}}_{\max}$ is the heat rate limit, and $C_{Q}$ is a constant parameter. ${\bar{N}}_{Z}$ and $n_{\max}$ are the normal load factor and its limit, and $\bar{q}$ and ${\bar{q}}_{\max}$ are the dynamic pressure and its maximum value.

If in a quasi-equilibrium glide condition (QEGC), the parameters retain the following relation [12,35]:

(6) $$\frac{L}{m}\cos\sigma_{\mathrm{QEGC}}-(g-\frac{V^{2}}{r})\geq 0$$

where $\sigma_{\mathrm{QEGC}}$ is the bank angle. This constraint reduces oscillations and preserves the bank angle margin. This is a soft constraint in the sense that its enforcement need not be too strict.

The re-entry vehicle is expected to reach a desired terminal condition, for which the state is specified by the altitude $h_{f}^{*}$ and velocity $V_{f}^{*}$ at a distance $R_{f}^{*}$ from the target location. The final state of re-entry is described by the final altitude $h_{f}$ and velocity $V_{f}$ at a distance $R_{f}$ from the target location, and the terminal constraints are given by

(7) $$V_{f}=V_{f}^{*},h_{f}=h_{f}^{*},R_{f}\;\leq \;R_{f}^{*}$$

The exponential atmosphere model [34] used for constraint transformation is:

(8) $$\rho =\rho_{0}e^{\left(-h/h_{r}\right)}$$

where $h=r-r_{e}$, $h_{r}$ is the atmospheric density scale height and $\rho_{0}=1.225\;\mathrm{kg}/m^{3}$.

The tracking is realized when the velocity vs. altitude plane in Equations (3)–(6) is transformed by replacing the density with the altitude in Equation (8) into

(9) $$h\geq h_{r}\ln(\frac{C_{Q}^{2}\rho_{0}V^{6.3}}{{\dot{Q}}_{\max}^{2}})$$

(10) $$h\geq h_{r}\ln\frac{C_{D}\rho_{0}V^{2}S(C_{L}/C_{D}\cos\alpha +\sin\alpha )}{2mgn_{\max}}$$

(11) $$h\geq h_{r}\ln\frac{\rho_{0}V^{2}}{2{\bar{q}}_{\max}}$$

(12) $$h\leq h_{r}\ln\frac{C_{L}\rho_{0}V^{2}S_{ref}\sigma_{\mathrm{QEGC}}}{2m(g-V^{2}/r)}$$

By analyzing the influence of the angle of attack (AoA) on the heat rate constraint, normal load factor constraint and dynamic pressure constraint, the shape of the AoA profile can be designed. In addition, as a result of the success of the path constraint management in the Shuttle strategy, an AoA profile can be pre-planned based on the nominal range-to-go, maximum L/D, etc. [3], with an example, as follows:

(13) $$\alpha_{profile}=\left\{ \begin{array}{cc} \alpha_{\max} & \left(V\geq V_{\alpha \_\max}\right)\\ k\times V+a & (V_{\alpha \_\min}\leq V<V_{\alpha \_\max})\\ \alpha_{L/D\max} & (V<V_{\alpha \_\min}) \end{array}\right.$$

where the maximum AoA $\alpha_{\max}$ is selected to avoid over-limit heat rate in rapid deceleration at initial re-entry, $\alpha_{L/D\max}$ is the AoA at maximum L/D for final glide, $V_{\alpha \_\max}$ and $V_{\alpha \_\min}$ are the maximum and minimum switch velocities, V is the current velocity, *k* represents the parameter of gradient between *α*_*L/D*_max__ and *α*_max_, and *a* denotes a compensation constant.

## 3. Skip Re-Entry Detection Solution

For low hypersonic L/D vehicles whose re-entry velocity is close to the escape velocity of 11.2 km/s, the Kepler phase often exists because of the high re-entry energy state. Conversely, high hypersonic L/D vehicles may not enter the Kepler phase without sufficient re-entry energy from low orbits. In this study, it is assumed that the skip re-entry of the vehicle is carried out in the atmosphere, and the detection of skip is focused on longitudinal motion.

### 3.1. Analysis on Phugoid Oscillation

As the equilibrium glide motion is performed at a baseline where $\dot{\gamma }\approx 0$ and the rotation rate of the Earth is neglected, the longitudinal state vector $x={\left[r\;V\;\gamma \right]}^{T}$ and the control vector u=cosσ are linearized [36] according to Equations (1), (2) and (9), as follows:

(14) $$\Delta \dot{x}=A\Delta x+B\Delta u$$

(15) $$A=\left[\begin{array}{ccc} 0 & \sin\gamma & V\cos\gamma \\ -\frac{D_{r}}{m}+\frac{2g\sin\gamma }{r} & -\frac{D_{V}}{m} & -g\cos\gamma \\ \frac{L_{r}\cos\sigma }{mV}-\frac{V\cos\gamma }{r^{2}}+\frac{2g\cos\gamma }{Vr} & \frac{L_{V}\cos\sigma }{mV}-\frac{L\cos\sigma }{mV^{2}}+\cos\gamma (\frac{1}{r}+\frac{g}{V^{2}}) & -\sin\gamma (\frac{V}{r}-\frac{g}{V}) \end{array}\right]$$

(16) $$B={\left[\begin{array}{ccc} 0 & 0 & \frac{L}{mV} \end{array}\right]}^{T}$$

where $D_{r}$, $D_{V}$, $L_{r}$, and $L_{V}$ are the partial derivatives. γ assumes small values in the re-entry process of equilibrium glide for medium and high *L/D* vehicles [37]. Then, the characteristic equations are the following:

(17) $$\begin{array}{l} \left|\lambda I-A\right|=\lambda^{3}+C_{1}\lambda^{2}+C_{2}\lambda +C_{3}=0\\ \left\{ \begin{array}{l} C_{1}=\frac{D_{V}}{m}\\ C_{2}=-\frac{L_{r}\cos\sigma }{m}+\frac{V^{2}}{r^{2}}+\frac{g}{V}\frac{L_{V}\cos\sigma }{m}\\ C_{3}=-\frac{D_{V}}{m}(\frac{2g}{r}-\frac{V^{2}}{r^{2}})+2\frac{D_{r}}{m}\frac{V}{r} \end{array}\right. \end{array}$$

According to Etkin [38] and Laitone and Chou [39], there are three modes for longitudinal motion at hypersonic speed, namely, the phugoid mode, short-period mode and spiral mode, and the root of the spiral mode is real and close to zero. Therefore, the “phugoid oscillation” mode is estimated by ignoring the $C_{3}$ term, as follows:

(18) $$\lambda^{2}+C_{1}\lambda +C_{2}=0$$

Then the “phugoid oscillation” frequency is approximated as

(19) $$\omega =\omega_{n}\sqrt{1-\zeta^{2}}=\sqrt{C_{2}-\frac{C_{1}^{2}}{4}}$$

and its corresponding spatial frequency is

(20) $$\Omega =\frac{\omega }{V}$$

**Figure 1:**
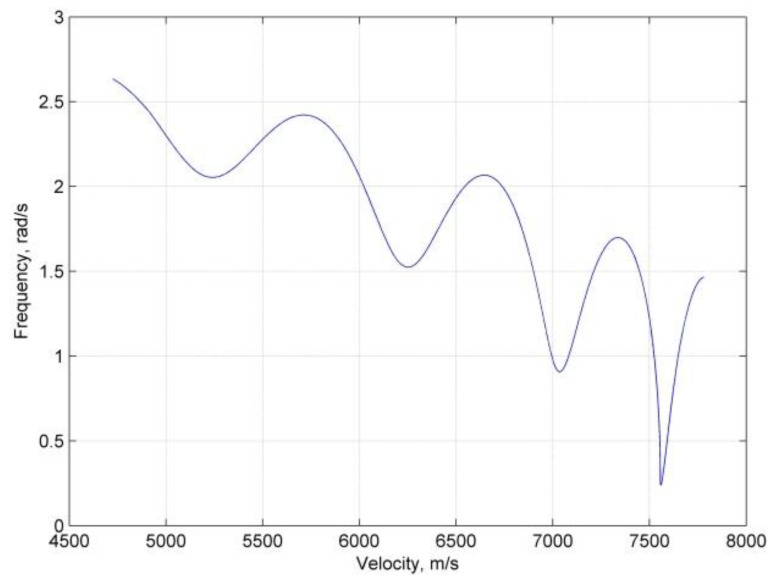
Typical frequency variation in skipping re-entry.

During the skips occurring at hypersonic Mach numbers, the AoA is given from the profile at $\alpha =\alpha_{\max}$, and the lift and drag coefficients are almost constant; therefore, the phugoid frequency changes with flight altitude and velocity. Figure 1 presents a typical frequency variation in a skipping re-entry. From the figure, it can be seen that the frequency is comparable to the ripple of the trajectory; furthermore, the frequency increases as the velocity decreases and shows additional oscillation with oscillations in the altitude. In view that the change in the frequency reflects the basic shape of the altitude vs. the velocity, an approximate skip altitude $h_{skip}$ is constructed with the piecewise frequency, as follows:

(21) $$h_{skip}=h_{\max}-F\Omega$$

where $h_{\max}$ is the predicted peak skipping altitude, and F is a constant factor determined according to the starting skip state, as follows:

(22) $$h_{0}=h_{\max}-F\Omega_{0},\;\;\;F=(h_{\max}-h_{0})/\Omega_{0}$$

where $h_{0}$ and $\Omega_{0}$ are the starting altitude and the corresponding spatial frequency, respectively.

**Figure 2:**
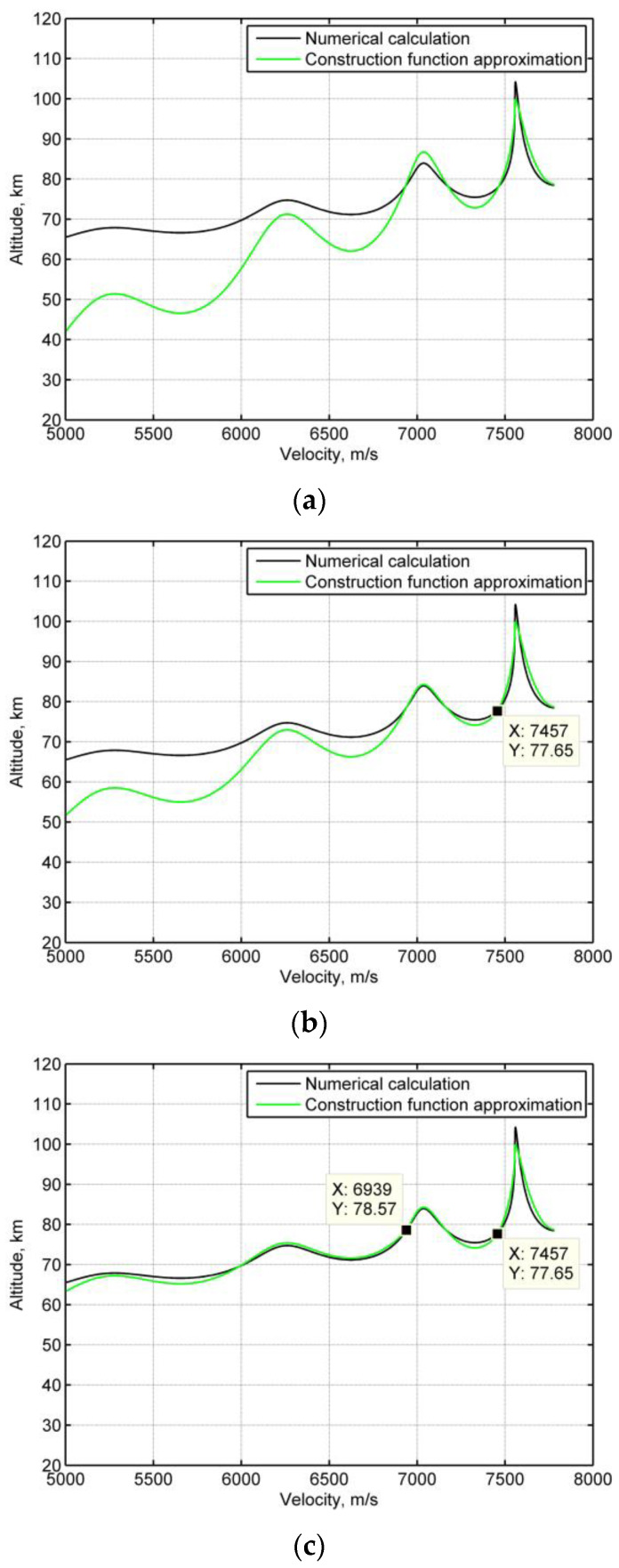
The phugoid oscillation motion trajectories by two prediction methods. (**a**) Fixed initial parameters, (**b**) initial parameters are updated for the second skip, (**c**) initial parameters are updated for the third skip.

Figure 2a presents the phugoid oscillation trajectories realized by constructing a function approximation from Equations (21) and (22) and numerical integration (the Runge–Kutta method is employed) as a baseline from Equations (1), (2), and (13). The construction function approximation matches the numerical integration, while the deviation of the construction function approximation accumulates with continuous skips. To reduce the deviation, the initial state parameters $h_{0}$, $\Omega_{0}$ and $h_{\max}$ in Equation (22) can then be updated at each skip. Figure 2b,c show that the accumulated deviation is significantly reduced for the second and third skip, if the initial state parameters are updated for these skips.

From the above results, the skip altitude can be estimated by the function of the current state in Equations (17)–(22).

### 3.2. Skip Detection Based on Estimated Skip Altitude

The residual deviation between the estimated skip altitude ($h_{skip}$) and the altitude output of the sensor measurement feedback (h) is used as the observation result in each period to detect the skip, that is:

(23) $$h_{k}=h-h_{skip}$$

Skip condition: $E[h_{k}]=0$. No-skip condition: $E[h_{k}]\neq 0$.

A means test is used for the residual evaluation for a selected skip velocity scope, and the analysis is performed in Section 5.2.1.

Establishing binary hypothesis:

$$H_{0}:\;\mathrm{Skip}\;H_{1}:\;\mathrm{No}-\mathrm{skip}$$

Define the false alarm rate $P_{F}=P(H_{1}\;/\;H_{0})$, missing detection rate $P_{M}=P(H_{0}\;/\;H_{1})$, and detection rate $P_{D}=1-P_{M}$. The designed skip re-entry detection function is:

(24) $$\lambda_{k}=w_{k}A_{k}$$

where $w_{k}$ is the weight coefficient and $A_{k}$ is the $h_{k}$ group mean. According to the central limit theorem, the mean value follows the normal distribution, and then the weighted data of the mean value follows the standard normal distribution, $\lambda_{k}∼N(0,\;1)$.

The skip is defined as

(25) $$\lambda_{k}\geq T_{D}$$

The no-skip is defined as

(26) $$\lambda_{k}<T_{D}$$

To determine $T_{D}$ according to the requirement of the system $P_{F}$:

(27) $$P_{F}=\alpha_{T}$$

then,

(28) $$P_{F}=\int_{-\infty }^{T_{D}}\frac{1}{\sqrt{2\pi }}e^{-\frac{x^{2}}{2}}d\lambda \;=\alpha_{T}\;\to T_{D}$$

where $\alpha_{T}$ is the expected value.

### 3.3. Skip Re-Entry Detection for Trajectory Control Logic

For vehicles with high hypersonic L/D, the tendency for “phugoid oscillation” in re-entry trajectory has been suppressed by augmenting guidance [13]. However, the oscillations have a range-increasing capability and can be used to extend the downrange.

The range rate is

(29) $$\dot{R}=V\cos\gamma$$

From Equations (1) and (2), Equation (29) is written as

(30) $$dR=\frac{V\cos\gamma }{-\rho V^{2}S_{ref}C_{D}/2m-g\sin\gamma +\Omega_{e}^{2}r\cos\phi (\sin\gamma \cos\phi -\cos\gamma \sin\phi \cos\psi )}dV$$

The hypersonic aerodynamic coefficients are almost constant with the Mach number at a specified AoA. As the flight path tends to be flat, e.g., in an altitude change of 40 km compared with a range of 2000 km, it is assumed that γ≈0 for the range estimation. To compare the range extending capability between quasi-equilibrium glide flights and skip flights, it is assumed that they are at the same latitude $\phi =0^{\circ }$. Then, Equation (30) can be rewritten as

(31) $$\tilde{R}\approx \frac{2m}{C_{D}S_{ref}}\int_{V_{2}}^{V_{1}}\frac{1}{\rho V}dV$$

where $V_{1}$ and $V_{2}$ are the specified velocities for concerned re-entry, normally $V_{\alpha \_\max}\leq V_{2}<V_{1}$.

It is shown that for a given vehicle and scheduled AoA profile, the atmospheric density is a main factor affecting re-entry range. In addition, by suppressing skips, $\cos\sigma_{\mathrm{QEGC}}<\cos\sigma_{skip}$, compared with the quasi-equilibrium glide flight, the skip motion at the same velocity will fly at a thinner atmospheric density, and thus ${\tilde{\rho }}_{\mathrm{QEGC}}>{\tilde{\rho }}_{skip}$. The quasi-equilibrium glide detours continuously by alternative banking and corresponds to a lower downrange than the skip:

(32) $$R_{\mathrm{QEGC}}<R_{skip}$$

**Figure 3:**
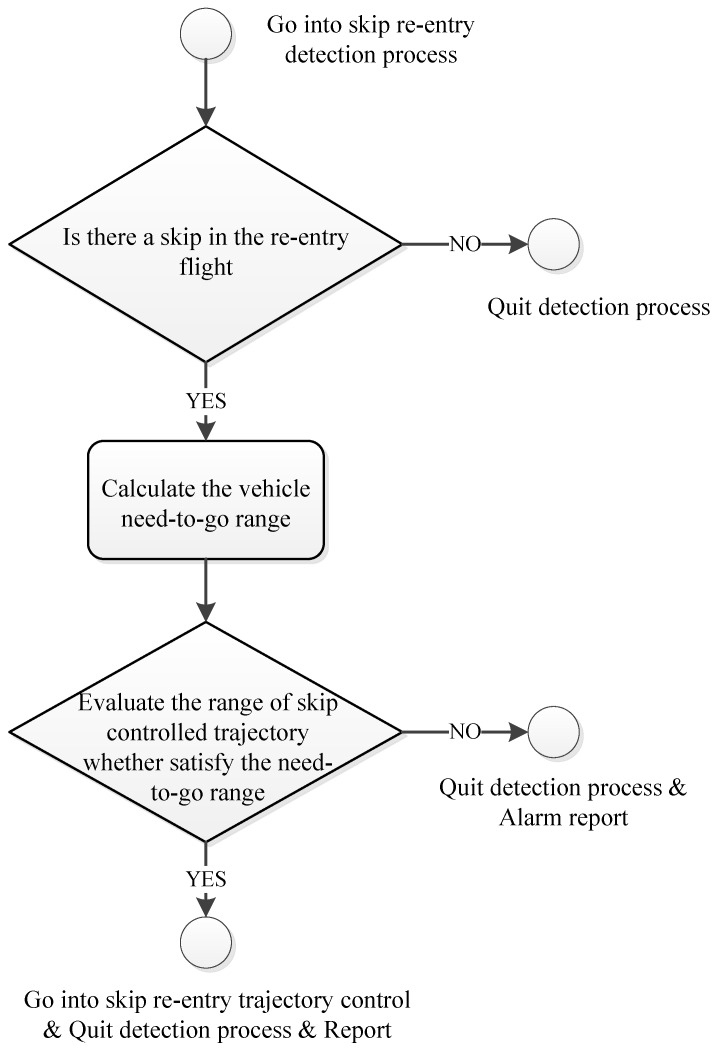
Skip detection for trajectory control logic.

After detecting the skip re-entry, the characteristics of the skip with increasing range can be used to guide the vehicle to the desired alternate landing area, and the skip range can be predicted by numerical integration from Equations (1), (2), (13), and (29). The transition logic from skip re-entry detection to trajectory control is shown in Figure 3.

## 4. Skip Re-Entry Trajectory Control

In this section, the implementation of the trajectory control by generating suitable bank angles when skip occurs is performed. The constraints are the heat rate, normal load factor and the dynamic pressure considered.

**Figure 4:**
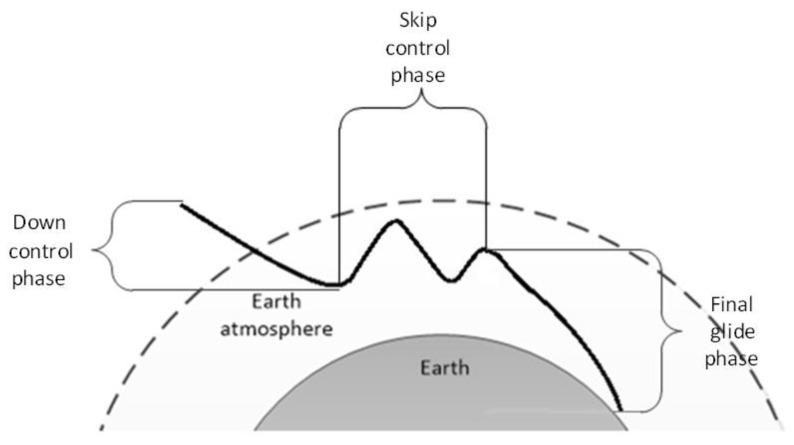
Skip re-entry sketch map.

The re-entry is normally divided into the down control phase and final glide phase [12]; however, once the skip occurs, the skip trajectory needs to be controlled. Therefore, in this study, the skip re-entry is divided into the down control, skip control, and the final glide phases, in a similar manner to the Apollo re-entry [14], as shown in Figure 4.

The down control phase is the same as in normal re-entry where the vehicle flies through vacuum into the atmosphere from the initial re-entry interface to the state when the descent rate is close to zero. During the down control phase, the bank angle $\sigma_{\mathrm{down}}$ is normally given by a constant which needs to satisfy boundary constraints [3].

The different bank angle control methods are used in skip control phase and final glide phase. With the α versus flight velocity profile given, a constant bank angle $\sigma_{skip}$ is employed in the skip control phase and reference trajectory-tracking method is used in the final glide. For the skip re-entry, the range threshold specified energy state is a reference condition, and the control phase transition logic is used for bank angle control method selection in the skip control phase and the final glide phase.

In normal re-entry, it is assumed that the quasi-equilibrium glide flight is employed for guidance, which suppress the skips. Therefore, we can obtain a skip guidance command magnitude constraint with the quasi-equilibrium glide flight condition [12], as follows:

(33) $$0\leq |\sigma_{skip}|<|\sigma_{\mathrm{QEGC}}|$$

In addition, when the AoA profile and the magnitude of constant $\sigma_{\mathrm{down}}$ are preset, the skip trajectory needs to satisfy the constraint boundary Equations (9)–(11). To predict the skip motion from a velocity–altitude profile, the Runge–Kutta numerical iteration method using Equations (1), (2), (13) and (9)–(11) can be employed to estimate the next skip state for a given $\sigma_{skip}$. Then, the maximum skip guidance command magnitude $\sigma_{skip\_\max}$ can be further identified within the dispersion tests of atmospheric density, lift and drag coefficients, and mass. Based on Equation (33), the skip guidance command magnitude constraint can be obtained as follows:

(34) $$0\leq |\sigma_{skip}|\leq |\sigma_{skip\_\max}|<|\sigma_{\mathrm{QEGC}}|$$

➣ Method 1: The reference trajectory-tracking algorithm

**Figure 5:**
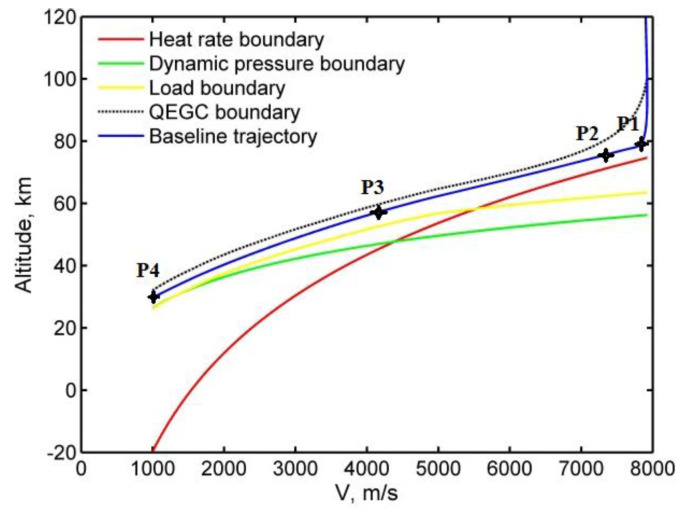
Reference baseline trajectory in the corridor of constraints.

To further satisfy the path constraints, a 3rd order reference baseline trajectory is designed for the final glide phase, as follows:

(35) $$h_{ref}=k_{3}V^{3}+k_{2}V^{2}+k_{1}V+k_{0}$$

where $k_{i}(i=0,1,2,3)$ are solved using reference points P1, P2, P3, and P4. P1 is the final point of the down control phase and P4 is the terminal constraint point, including the final velocity and altitude conditions. P2 is the state point with zero descent rate after the first skip that can be predicted based on the Runge–Kutta method. P3 is a designed point between P2 and P4 to construct the reference baseline trajectory. All the points are needed to satisfy the velocity–altitude path constraints profile, as shown in Figure 5. The skip altitude for the skip motion is higher than that of the reference baseline trajectory at the same velocity; this further proves that the skip satisfies path constraints.

To track the reference baseline trajectory and obtain the behavior of a stable second order feedback system in altitude deviation, Equation (1) is rewritten as:

(36) $$\dot{h}=V\sin\gamma$$

and,

(37) $$\ddot{h}=V\dot{\gamma }\cos\gamma +\dot{V}\sin\gamma$$

Because $h_{ref}$ belongs to the velocity–altitude profile Equations (9)–(12), it satisfies the QEGC condition $\dot{\gamma }\approx 0$, and flight path tends to be flat, e.g., an altitude change of 60 km compared with a range of 8000 km. Thus, $\Delta \dot{h}=\dot{h}-{\dot{h}}_{ref}\approx \dot{h}$ and $\Delta \ddot{h}=\ddot{h}-{\ddot{h}}_{ref}\approx \ddot{h}$, and the altitude deviation feedback control law can be designed as the following,

(38) $$\ddot{h}+2\xi_{h}\omega_{h}\dot{h}+\omega_{h}{}^{2}(h-h_{ref})\approx 0$$

where the parameter $\omega_{h}$ and $\xi_{h}$ are the designed undamped natural frequency and damping. Inserting Equations (1), (36), and (37) into Equation (38) leads to the following expression:

(39) $$\cos\sigma_{final}=\frac{mg}{L}-\frac{mV^{2}r}{L}-\frac{\left(2\xi_{h}\omega_{h}V\sin\gamma +\omega_{h}{}^{2}\Delta h\right)}{\cos\gamma }$$

From the above description, the velocity vs. altitude profile can be used as a monitor. Then, the minimum magnitude constraint of guidance command can be obtained when Equation (6) takes the minimum value by $\sigma_{EQ}$, and the maximum magnitude constraint needs to satisfy the path constraints. Hence, we have

(40) $$\left|\sigma_{EQ}|\leq |\sigma_{final}|<|\sigma_{path}\right|$$

where $\sigma_{path}$ is the bank angle according to the path constraints and can be obtained using the path constraints velocity and altitude in equilibrium glide condition, where $\dot{\gamma }\approx 0$ and the rotation rate of the Earth is neglected:

(41) $$\frac{L}{m}\cos\sigma_{path}-(g-\frac{V_{path}^{2}}{h_{path}+r_{e}})\;\approx \;0$$

Therefore, if $\sigma_{final}$ is beyond the boundary, a preset boundary value satisfying the Equation (40) will be employed for guidance command.

➣ Method 2: The control phase transition logic

Based on the range-to-go, the skip trajectory is adjusted through control phase transition logic, including the selection of tracking $h_{ref}$ and employing skips by the threshold range $R_{thres}$, as follows:

(42) $$\begin{array}{l} \sigma =(1-\omega_{0})\sigma_{skip}+\omega_{0}\sigma_{final}\\ \omega_{0}=\left\{ \begin{array}{ll} 0, & R_{togo}>R_{thres}\\ 1, & R_{togo}\leq R_{thres} \end{array}\right. \end{array}$$

and,

(43) $$R_{thres}\approx R_{\mathrm{QEGC}}-\sum_{V_{1}}^{V_{2}}R(\alpha_{profile},\sigma_{final}),\begin{array}{cc} & V_{\alpha \_\max}\leq V_{2}<V_{1} \end{array}$$

**Figure 6:**
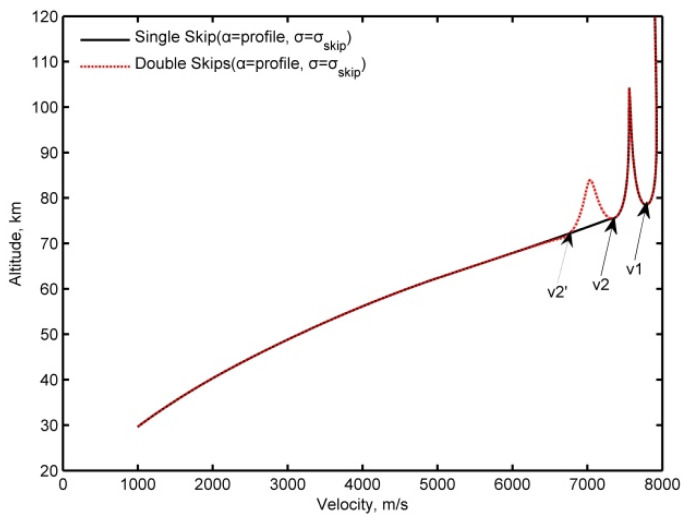
Skips and baseline trajectory combination.

The range threshold is the boundary to decide the transition from the skip control phase to the final control phase depending on the skip ending velocity $V_{2}$, as shown in Figure 6. The interval from $V_{1}$ to $V_{2}$ is employed for “phugoid oscillation”, and the range boundary $R_{thres}$ is approximately converted into the accumulated range after tracking $h_{ref}$ from $V_{2}$ to the end. $V_{1}$ is the velocity at the state when the descent rate is close to zero, $V_{\alpha \_\max}$ is a designed velocity in Equation (13). The range threshold state is specified as the related altitude $h_{thres}^{}$ and velocity $V_{thres}^{}$ at a distance $R_{thres}$ from the target location.

➣ Method 3: The lateral guidance logic

For the lateral guidance logic, the sign of the bank angle is determined by the threshold for bank angle reverse, which is used during the flight phases. In the skip control phase, the reverse threshold is the deviation of the cross range. In the final glide phase, the deviations of the cross range and course angle are employed to determine the sign of the bank angle for different distance conditions, as follows:

(44) $$\left\{ \begin{array}{ll} |\Delta c_{r}|\leq \Delta c_{thres} & R_{togo}\geq R_{course}\\ |\Delta \psi |\leq \Delta \psi_{thres} & R_{togo}<R_{course} \end{array}\right.$$

where $\Delta c_{r}$ and Δψ are the deviations of the cross range and course angle, respectively. $\Delta c_{thres}$ and $\Delta \psi_{thres}$ are the respective deviation thresholds of the cross range and course angle, $\Delta c_{thres}$ is designed based on the velocity function, and the course angle is controlled by entering the range boundary $R_{course}$. $R_{togo}$ is the distance between the current position of the vehicle and the destination position. When the deviation is higher than the threshold, the bank angle sign is changed. The Δ*ψ_thres_* selecting principle is mainly based on the terminal course angle constraint, and the $\Delta c_{thres}$ choosing rule is determined based on the bank angle reversal frequency.

In this paper, $\sigma_{skip}$ is a main variable and is used for adjusting the extension range in the re-entry process. To establish a skip re-entry trajectory control, the range threshold specified states, $V_{thres}^{*}$, $h_{thres}^{*}$, and $e_{thres}^{*}=\frac{1}{2}V_{thres}^{*2}+gh_{thres}^{*}$, are preset in a way to satisfy the final re-entry condition (the footprint method can be used to design and evaluate the specified state [40]), and then $\sigma_{skip}$ is calculated to guide the vehicle to the desired range threshold state.

The $\sigma_{skip}$ numerical search algorithm is presented in Algorithm 1.
**Algorithm 1.** The motion model-based $\sigma_{skip}$ numerical search algorithm.**Input**: Current re-entry motion state of the vehicle **Output:**$\sigma_{skip}$ **Control variable:**α, σ 1: *Calculate the range-to-go*$R_{togo}$ 2: *Load Equation (1) state and parameters.* 3: *Load expected range threshold state:*$e_{thres}^{*}(V_{thres}^{*},h_{thres}^{*})$. 4: **for** [${\hat{\sigma }}_{skip}=0\;\mathrm{to}\;\sigma_{skip\_}{}_{\max}$(increase 5 degrees per cycle) ] **do** 5:  **for** (i=0 to N) **do** 6:   *3-DOF equations numerical integration output assignment* 7:    *Compare*
$R_{thres}$
*and*
$R_{togo}$*, call* Method 2; 8:   **if**
$R_{thres}<R_{togo}$
**then** 9:     $\alpha =\alpha_{profil}$, $\sigma ={\hat{\sigma }}_{skip}$, *call* Method 3; 10:   **else**
11:     *call* Method 1; 12:     $\alpha =\alpha_{profil}$, $\sigma =\sigma_{final}$, *call* Method 3; 13:   **end** 14:   α, σ
*input to 3-DOF equations and numerical integration*; 15:   **if**
$R_{togo}\leq R_{thres}$
**then** 16:     *Calculate the current state*
$e_{thres}(V_{thres}^{},h_{thres})$; 17:     **break;** 18:   **end** 19:  **end** 20: **end** 21: *According to*
${\hat{\sigma }}_{skip}$
*given and*
$e_{thres}(V_{thres}^{},h_{thres})$, $e_{thres}(V_{thres}^{},h_{thres})$
*and*
${\hat{\sigma }}_{skip}$
*database is built, and*
$e_{thres}\;\mathrm{vs}.\;{\hat{\sigma }}_{skip}$
*curve is fitted by the least square method;* 22: *Input*
$e_{thres}^{*}(V_{thres}^{*},h_{thres}^{*})$
*and calculate*
${\hat{\sigma }}_{skip}^{*}\;$; 23: $\sigma_{skip}={\hat{\sigma }}_{skip}^{*}\;$.

To form skip re-entry guidance, α and σ are the control variables in each guidance cycle, while α is provided by the profile represented by Equation (13). The calculation of σ depends on different guidance strategies of Equations (39), (42), (43) and (44) and the $\sigma_{skip}$ numerical search algorithm for each flight phase. During the down control phase, $\sigma_{skip}$ can be predicted to prepare for skip motion.

When a skip re-entry is detected, the above numerical search algorithm can be used to correct $\sigma_{skip}$ according to the current state in a designed period. At the final glide phase, the reference trajectory-tracking method is employed to enhance the robustness.

## 5. Simulations and Results

### 5.1. Example Vehicle and Preset Parameters

The example vehicle is taken to be similar to the Space Shuttle [41], with a mass of 76,297 kg and a hypersonic lift-to-drag ratio of 1.884 at a Mach number of 20 and an angle of attack of 40°. The pre-planned AoA profile corresponds to Equation (13), as follows:

$$\alpha_{profile}=\left\{ \begin{array}{ll} 40^{{}^{\circ}} & \left(V\geq 5000\;m/s\right)\\ 0.00625V+15 & \left(1000\;m/s\leq V<5000\;m/s\right)\\ 15^{{}^{\circ}} & \left(V<1000\;m/s\right) \end{array}\right.$$

When the flight vehicle returns to the atmosphere from a low Earth orbit, the deorbit impulse and transfer orbit are mainly calculated according to the given re-entry window into the atmosphere (including initial re-entry altitude, velocity, and flight path angle). Therefore, the assumed initial longitude and latitude are given in this paper, the initial re-entry altitude, velocity, and flight path angle are provided according to references [42,43], and the mission conditions are listed in Table 1.

### 5.2. Skip Re-Entry Detection

#### 5.2.1. Mean Test

**Figure 7:**
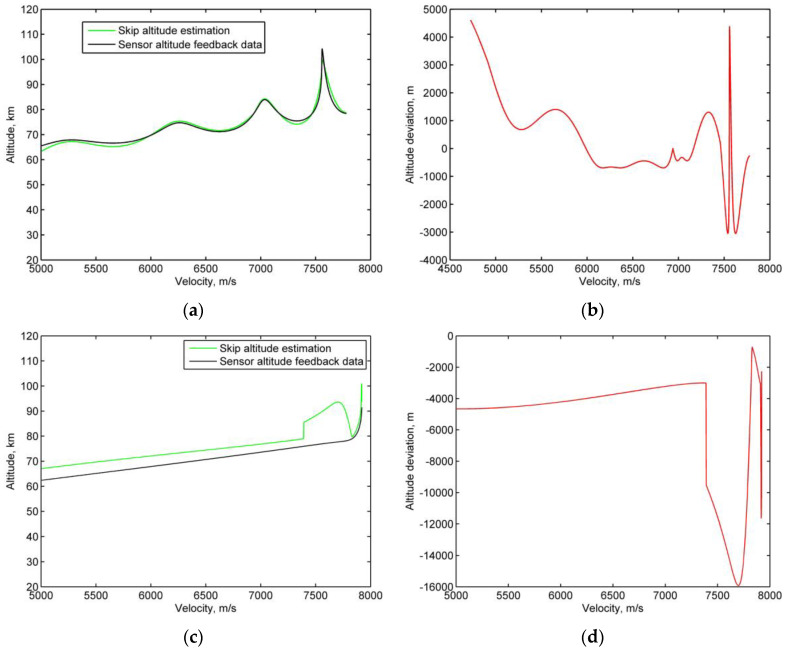
Comparison of the residuals between the skip re-entry and the quasi-equilibrium glide re-entry. (**a**) Skip altitude estimation vs. the sensor altitude feedback data in skip re-entry; (**b**) skip re-entry residuals between the skip altitude estimation and the sensor altitude feedback data; (**c**) skip altitude estimation vs. the sensor altitude feedback data during quasi-equilibrium glide re-entry; (**d**) quasi-equilibrium glide re-entry residuals between the skip altitude estimation and the sensor altitude feedback data.

In this study, residual deviations between the estimated skip altitude and the altitude output of the sensor measurement feedback are compared in each period and used as a skip re-entry detection condition. Figure 7b,d show the mean residual deviations between the estimated skip altitude and the sensor feedback data. Because the altitude of the skip is generally higher than that of quasi-equilibrium glide, Figure 7b shows a small mean residual deviation when a skip re-entry happens; however, Figure 7d shows a significant negative deviation when the re-entry is in quasi-equilibrium glide. Therefore, a mean threshold was used as the decision-making condition to unambiguously distinguish the skip re-entry state from the no skip re-entry state.

#### 5.2.2. False Alarm Rate and Detection Rate Test

**Figure 8:**
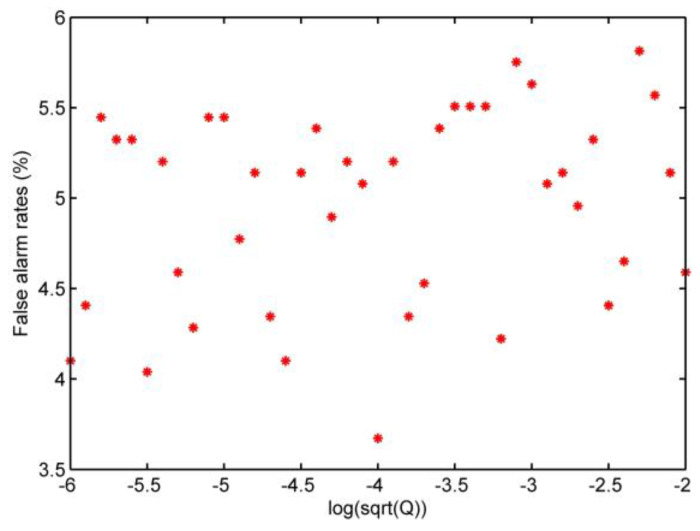
False alarm rates test.

Based on the description in Section 3.2, the false alarm rate and the detection rate of the skip re-entry are defined as $P_{F}$ and $P_{D}$. In this study, it is assumed that the process noise is zero mean white noise with a standard deviation of $Q^{1/2}$, and the standard deviation of the noise changes from $1\times 10^{-6}$ to $1\times 10^{-2}$. Each simulation generated 500 data points, and every five consecutive data points form a group to calculate the mean value. To design $w_{k}=1/1800$, a false alarm rate $\alpha_{T}=8\%$, and from the look-up table $T_{D}=-1.41$ is employed. The test results for the false alarm rate are shown in Figure 8.

Figure 8 shows that the false alarm rate varies from 3.5% to 6%, suggesting that the threshold determined by the formula can meet the requirement $\alpha_{T}=8\%$.

**Figure 9:**
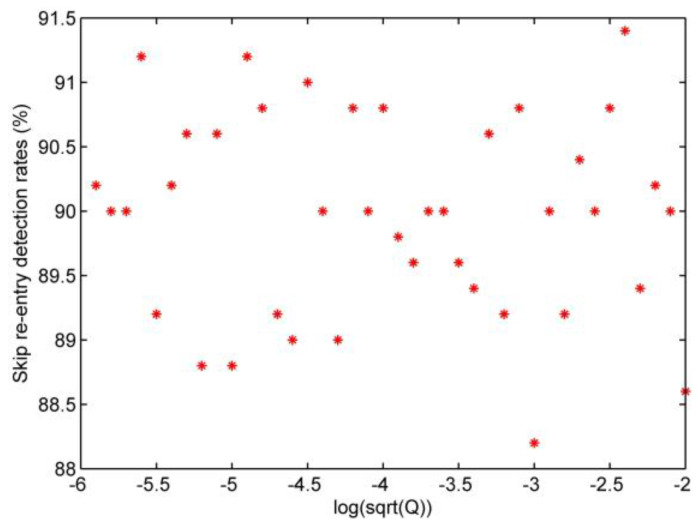
Skip re-entry detection rates test.

Figure 9 shows that when the above values of the threshold and noise interference parameters are used in this detection rate test, the skip re-entry detection rate varies from 88% to 91.5%.

### 5.3. Skip Re-Entry Trajectory Control Test

#### 5.3.1. Execution Time Performance Test of the $\sigma_{skip}$ Numerical Search Algorithm

**Figure 10:**
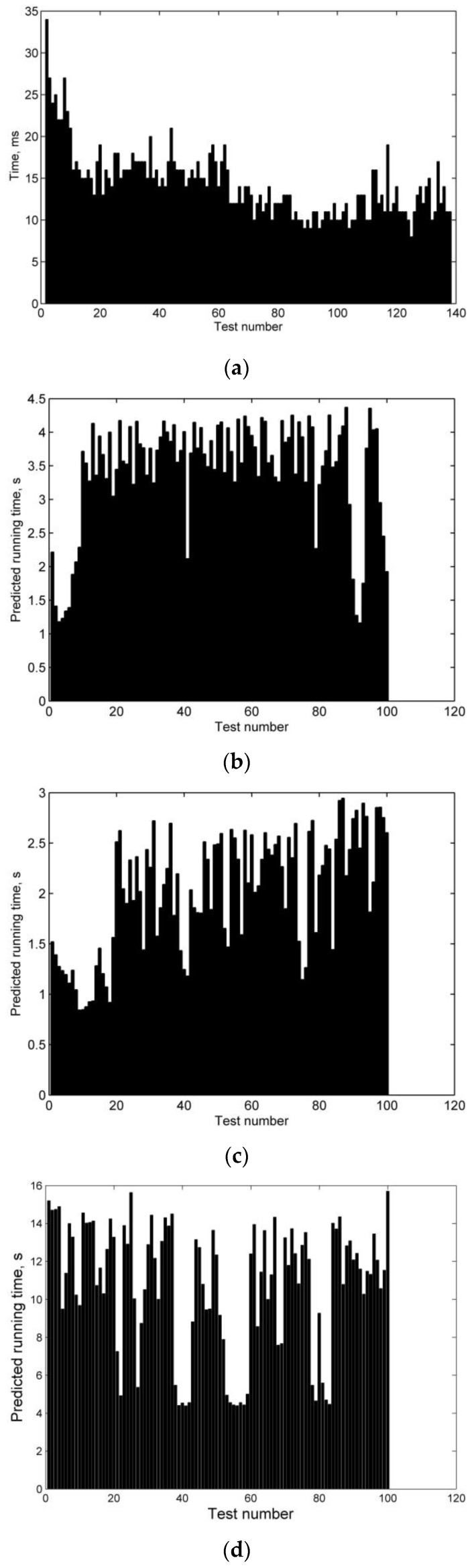

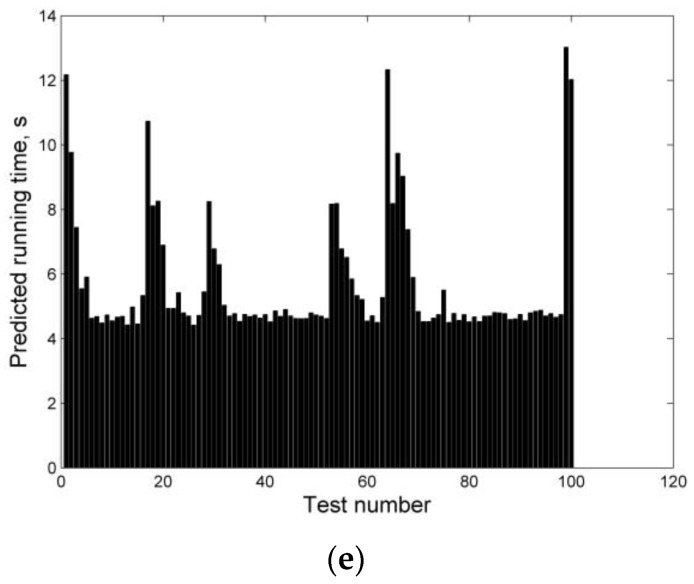
Running time tests for the $\sigma_{skip}$ numerical search algorithm. (**a**) Each motion state calculated time test, (**b**) Each ${\hat{\sigma }}_{skip}$ given calculated time test from initial re-entry state, (**c**) Each ${\hat{\sigma }}_{skip}$ given calculated time test from skip re-entry state, (**d**) $\sigma_{skip}$ searching calculated time test from initial re-entry state, and (**e**) $\sigma_{skip}$ searching calculated time test from skip re-entry state.

This section is used to evaluate the running time for the $\sigma_{skip}$ numerical search algorithm from three aspects: each motion state calculation, each ${\hat{\sigma }}_{skip}$ given calculation from current state to the expected range threshold, and $\sigma_{skip}$ searching calculation, as shown in Figure 10a–e.

The computer configuration is an Intel(R) Core(TM) i5-4200H CPU @ 2.80GHz, RAM 4.00GB, Windows 8 operating system.

Figure 10a shows that the algorithm requires less than 35 ms to recur from the current state to the next state through a numerical integration of the 3-DOF equations using the RK4 method, which can meet the calculation requirements of the on-board computer within a period of 40 ms. Figure 10b,c are the calculation times of the algorithm cycle after the assumed bank angle command ${\hat{\sigma }}_{skip}$ is given at different positions. Figure 10b is the calculation from the re-entry position to the desired range threshold $R_{thres}$, and Figure 10c is the calculation from the skip detection position to the expected range threshold $R_{thres}$. By comparing the two figures, it can be seen that the farther the distance from the current position of the vehicle to $R_{thres}$ is, the longer the calculation time of the algorithm will be. Figure 10b shows that the calculation time of the algorithm is less than 4.5 s, while that of Figure 10c is less than 3 s. Figure 10d,e are the times for the algorithm to calculate the skip bank angle command based on the distance from the current position of the vehicle to the range threshold $R_{thres}$. Figure 10d shows the calculation times for the current position of the vehicle at the down control phase of re-entry, and Figure 10e shows the calculation times for the current position at the skip control phase. The comparison between the two figures shows that the average calculation time of the former is about 10 s, while that of the latter is about 5 s, which shows that the algorithm’s online calculation time is shorter when the vehicle approaches to the range threshold. This suggests that the algorithm supports the requirement that the online correction time decreases with decreasing range-to-go.

#### 5.3.2. Skip Re-Entry Trajectory Control Test under Monte Carlo Method

The mission conditions are listed in Table 1. For the 1000 trials of the Monte Carlo analysis, Gaussian-shaped or uniform dispersions are assumed, as summarized in Table 2. The analytical atmospheric density dispersion with respect to the 1976 U.S. Standard Atmosphere is used to imitate atmospheric uncertainties [44].

This paper focuses on detecting the unexpected skip re-entry and guiding the vehicle to the planned recovery area in case of an emergency. The terminal conditions are as follows:

$$V_{f}^{*}=1000\;m/s\;(\pm 2\%)\;h_{f}^{*}=30\;\mathrm{km}\;(\pm 5\%)\;R_{f}^{*}=30\;\mathrm{km}$$

If the ending position satisfies the velocity and altitude requirements and is within the precision circle of a radius of 30 km, the mission is considered to be a success; beyond the circle of radius of 50 km, it is considered to be a failure, and if in between, it is counted as a 50% success.

**Figure 11:**
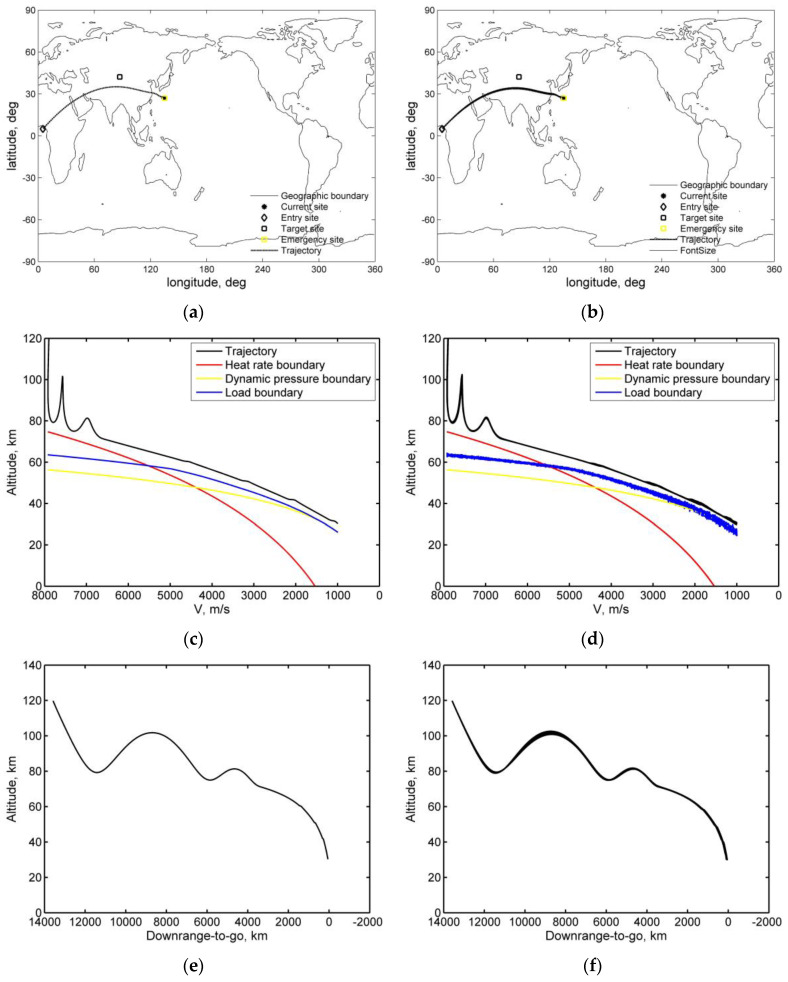

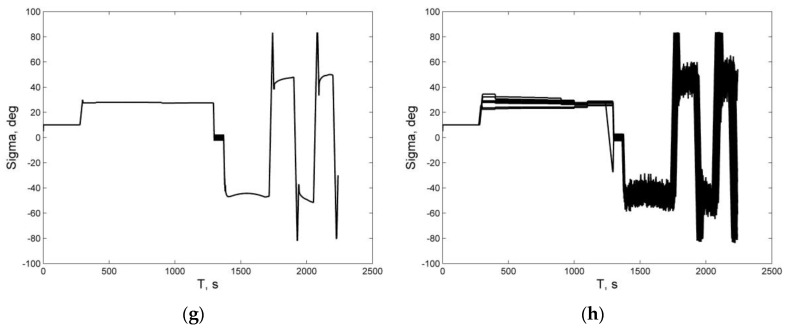
Simulations under Monte Carlo dispersions in a skip re-entry: (**a**) skip re-entry lateral nominal trajectory, (**b**) skip re-entry lateral histories, (**c**) nominal velocity versus altitude trajectory, (**d**) velocity versus altitude histories, (**e**) nominal downrange trajectory, (**f**) downrange histories, (**g**) nominal bank angle, (**h**) bank angle histories.

Figure 11 shows the histories under Monte Carlo dispersion (a subset of 1000 simulations). The skip re-entry detection threshold and parameter set is the same as in Section 5.2.2., the initial state of re-entry is given by the parameters of mission 1 in Table 1, the roll rate is less than $5^{{}^{\circ}}/s$, the range threshold is preset to $R_{thres}=4000\;\mathrm{km}$, the range threshold specified state is $e_{thres}^{*}(\;V_{thres}^{*}=6846\;m/s,\;h_{thres}^{*}=78,000\;m)$, and $\sigma_{skip}\in [-50^{{}^{\circ}},\;50^{{}^{\circ}}]$. During the skip control phase, the bank angle correction period is 100 s.

Figure 11a,c,e,g illustrates nominal histories, while Figure 11b,d,f,h shows the histories of state variables (30 examples from 1000 trials) under Monte Carlo dispersions to show the robustness of the skip trajectory control algorithm. The normal re-entry plan for a vehicle is to fly from the initial re-entry positon to the target site; when the skip re-entry is detected, the vehicle can be operated in accordance with the standby emergency plan to turn to the emergency site for recovery. In the down control phase, $\sigma_{skip}$ numerical search algorithm is used to predict the bank angle command in the case of skip re-entry. In the skip control phase, the algorithm is used to correct the energy needed to reach the range threshold, and the algorithm call cycle is 100 s in these tests. There are two elements that are specifically dedicated to the robustness of the algorithm, the first of which is the correction of bank angle in the skip control phase to satisfy the range threshold expected energy, and the second one is involved in the reference trajectory tracking law in the final glide phase to restrain the influence of process disturbances.

**Figure 12:**
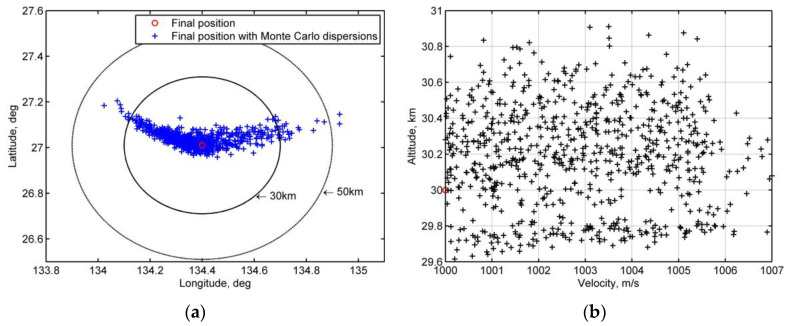
Final position deviation between nominal re-entry and re-entry with Monte Carlo dispersion (1000 trials): (**a**) final position deviations (**b**) velocity and altitude deviations.

Figure 12a shows the terminal miss distances for all tests, in which the terminal points within a circle of a radius of 30 km are assumed to be successful in view of the next stage of terminal area guidance, and the points within the circles of radius 30–50 km are considered to be semi-successful. For the 1000 trial simulations with parameter dispersions, the success rate is above 90%. In addition, when the vehicle arrives at the final re-entry velocity condition, Figure 12b shows that the altitude deviation varies from 29.6 km to 31 km, which satisfies the ±5% requirement.

The main purpose of this emergency solution is to recover the vehicle, and the velocity of the vehicle arriving at the position is 1000 m/s. Under this condition, the vehicle can hover and descend to help the ground find its position.

### 5.4. Skip Re-Entry Detection and Trajectory Control Application

#### 5.4.1. The Proposed Solution in Abnormal Skip Re-Entry Emergency Scenarios

**Figure 13:**
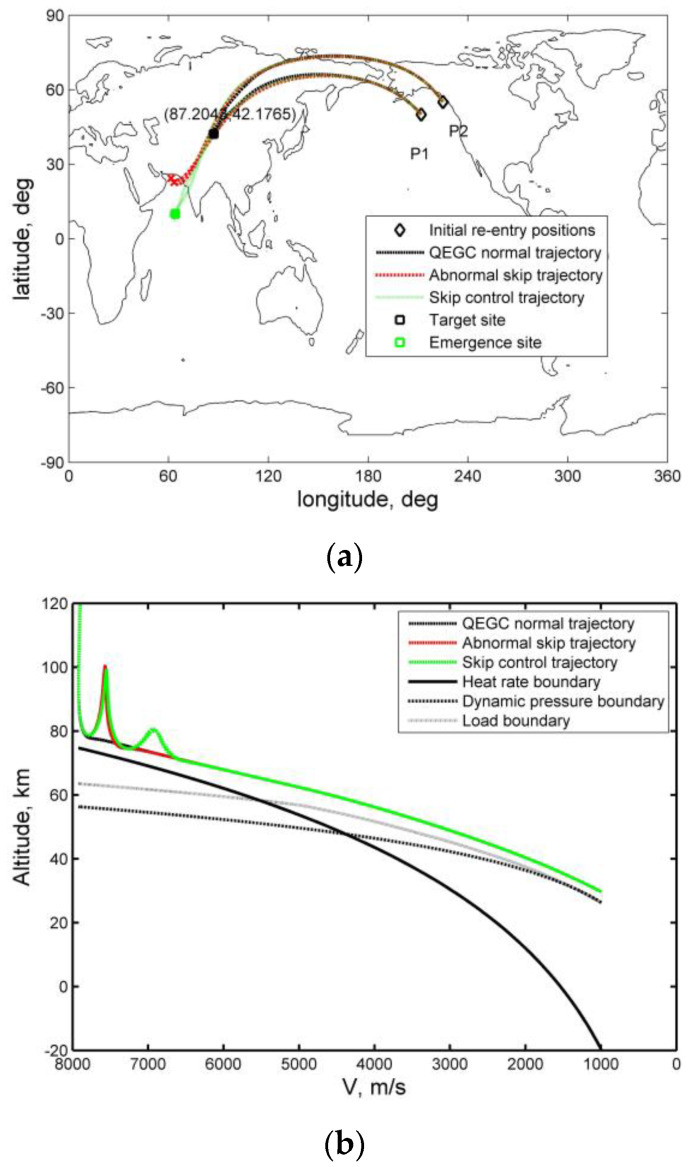
Emergency re-entry of a maneuvering vehicle for normal and abnormal trajectory control: (**a**) lateral histories for normal and abnormal re-entry trajectories, (**b**) velocity–altitude histories for normal and abnormal re-entry trajectories.

Figure 13 shows the cases of emergency re-entry of a maneuvering vehicle along an abnormal skip trajectory. The vehicle carries out a planned QEGC normal re-entry from the sites P1 and P2, respectively. However, due to a degradation of sensor performance, the vehicle does not enter the QEGC control mode due to the conditions such as “the total aerodynamic acceleration is greater than or equal to 1.52 m/s^2^” in reference [13] or “the load is greater than 0.05 g” in reference [23], but still flies according to the guidance commands of the capture atmosphere, and the vehicle flies along the abnormal skip trajectory. If the QEGC control algorithm is not called, there is a delay in calling it or is not suitable for skip trajectory control, the vehicle may be out of control, which threatens the safety of the ground. Possible points of failure are indicated by a red “x”, as shown in Figure 13a. In order to reduce the risk after such a situation occurs, the proposed solution for skip re-entry detection and trajectory control can be used as a reference method to guide the vehicle to the emergency area, as shown in Figure 13a,b.

#### 5.4.2. The Application of QEGC Method and the Proposed Solution in Abnormal Skip Re-Entry Scenarios

**Figure 14:**
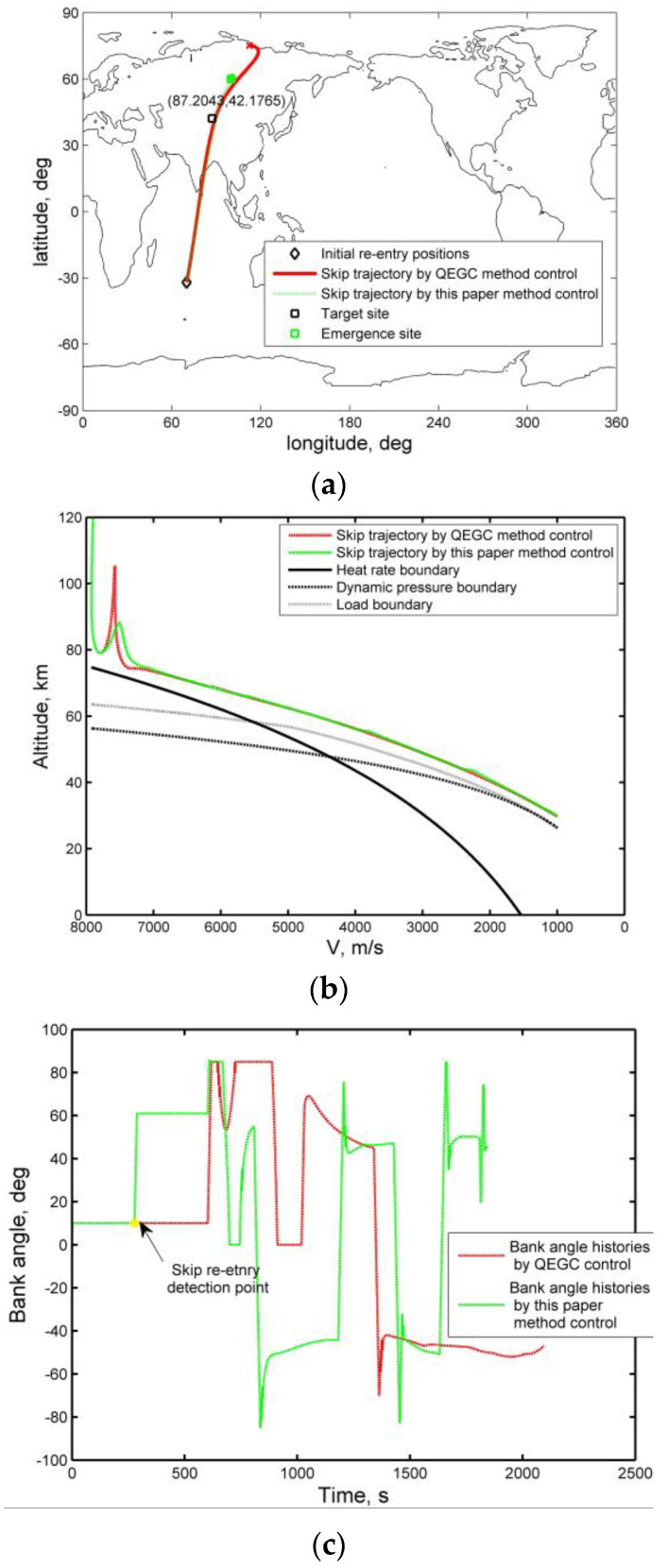
Comparison of quasi-equilibrium glide condition (QEGC) method and the proposed solution in an abnormal skip re-entry scenario: (**a**) lateral histories, (**b**) velocity–altitude histories, and (**c**) bank angle histories.

This section is devoted to the analysis of the influence of the QEGC method and the method presented in this paper on the trajectory control of skip re-entry. Assuming that the performance of the sensor is degraded, QEGC control is called with a delay, the bank angle continues to use the down control phase command, as shown in Figure 14c, and thus there will be a skipping phenomenon. The method described in this paper, with the function of skip detection, can help to drive the vehicle to the desired alternate area. However, QEGC method is no longer suitable for skip re-entry control, which makes it impossible to smoothly control the vehicle to an alternate area, as shown in Figure 14a.

Generally, QEGC re-entry is based on the designed nominal trajectory, and it contains no detection function for an unexpected skip trajectory. As shown in Figure 14b, in the process of hypersonic re-entry, when the skip occurs, the delayed call of the QEGC algorithm is likely to increase the short-term skip amplitude. Though the original QEGC algorithm emphasizes the suppression of a skip, and it may be successful in suppressing the skip within a certain period of time, the vehicle would by then have deviated too far from its nominal trajectory, which may lead to a failure of the re-entry mission.

The solution proposed in this paper adds to the detection function of skip re-entry, which can utilize the extended range abilities generated by a skip. When an unexpected skip occurs, it can actively use the skip ability to guide the vehicle to the desired recovery area and satisfy the path constraints.

## 6. Discussion

For the skip trajectory control, a range threshold $R_{thres}$ also can be found by referring to flight history data. In addition, to improve the accuracy of the final position for future work, the increase in the correction frequency of a given skip command and the improvement of the guidance algorithm in the final glide phase will be studied. The final re-entry condition in this paper refers to the energy management window condition. In future work, the question of how to deal with the area guidance problem of emergency alternate terminal will be further studied.

## 7. Conclusions

This paper is dedicated to the detection of unexpected skip re-entry and active use of the natural phugoid oscillations of medium or high L/D re-entry of the vehicle to guide it to the emergency area for recovery. The following conclusions can be drawn.

(1) An approximate analytical relationship is constructed for skip altitude estimation based on the oscillation frequency characteristic of the linearized re-entry motion equation of the vehicle. Based on the above analytical relationship, the skip re-entry detection method can be used as a standby tool of airborne monitoring in the form of software to alert the skipping during the re-entry process or to prepare to call other emergency trajectory control strategies;
(2) Based on the pre-planned angle of attack profile, a phase separation of the down control, skip control, and final glide phases is employed in this paper. On this basis, a control phase transition logic-based on the range threshold under the velocity–altitude profile is proposed, which can smoothly connect the skip control phase and final glide phase, and provide support in calling related guidance algorithms at each phase to achieve a single or multiple skip re-entry to improve range capabilities;
(3) Simulations further demonstrate that the proposed solution can achieve an expected detection rate, its running time is reasonable, and the trajectory control can satisfy path constraints and be robust under Monte Carlo dispersions. Finally, it has also been demonstrated that the method can guide the vehicle to an emergency area for recovery when it skips.

## Figures and Tables

**Table 1:** Mission parameters.

Parameters	Mission1	Mission1	Mission2	Mission3
Initial altitude of re-entry, km	120	120	120	120
Initial longitude of re-entry, deg	5	212	225	70
Initial latitude of re-entry, deg	5	50	55	−32
Initial Earth-relative velocity of re-entry, m/s	7900	7900	7900	7900
Initial flight path angle of re-entry, deg	−1.5	−1.5	−1.5	−1.5
Longitude of target site, deg	87.2043	87.2043	87.2043	87.2043
Latitude of target site, deg	42.1765	42.1765	42.1765	42.1765
Terminal altitude, km	30	30	30	30
Terminal velocity, m/s	1000	1000	1000	1000
Longitude of emergency site, deg	134.4	64	64	100
Latitude of emergency site, deg	27	10	10	60

**Table 2:** Dispersions of re-entry interface state and other parameters.

Parameters	Distribution	Max
Re-entry initial longitude, deg	Gaussian	0.1
Re-entry initial latitude, deg	Gaussian	0.1
Re-entry initial relative velocity, m/s	Gaussian	5
Re-entry initial flight path angle, deg	Gaussian	0.05
Re-entry initial heading angle, deg	Gaussian	0.5
*C_L_*	Gaussian	20%
*C_D_*	Gaussian	20%
Atmospheric density	Analytical	30%
Mass, kg	Uniform	±1%
